# A Pilot Study on Serial Nerve Ultrasound in Miller Fisher Syndrome

**DOI:** 10.3389/fneur.2020.00865

**Published:** 2020-08-14

**Authors:** Hsueh-Wen Hsueh, Kai-Chieh Chang, Chi-Chao Chao, Sung-Tsang Hsieh

**Affiliations:** ^1^Department of Neurology, National Taiwan University Hospital, Taipei, Taiwan; ^2^Department of Neurology, National Taiwan University Hospital Yunlin Branch, Yunlin, Taiwan; ^3^Graduate Institute of Anatomy and Cell Biology, National Taiwan University Hospital College of Medicine, Taipei, Taiwan

**Keywords:** Miller Fisher syndrome, nerve sonography, facial nerve, Guillain-Barre syndrome, follow-up

## Abstract

**Objective:** Miller Fisher syndrome (MFS) is predominantly a clinical diagnosis, with classic triad of ophthalmoplegia, ataxia, and generalized reduced reflexes. Previous studies in chronic and acute immune-mediated neuropathies indicated that ultrasound, may help to detect changes that could correspond with disease activity. We studied the feasibility of serial nerve ultrasound in MFS, using a healthy controls.

**Methods:** All MFS patients (*n* = 5) and healthy controls (*n* = 18), underwent a standardized sonographic protocol that evaluated nerve sizes of facial, large arm and leg nerves, and spinal nerve roots. All MFS patients underwent routine ancillary investigations, including electrodiagnostic testing and for presence of anti-GQ1b antibodies. In addition, four MFS patients had 2nd, and 3rd clinical and sonographic evaluation at 14 and 90 days from onset.

**Results:** The width of the facial nerve was significantly larger in the MFS group than in the control group (MFS: 1.19 ± 0.31 mm vs. normal: 0.67 ± 0.13 mm, *P* = 0.01). The size of the cervical roots and the nerves in the limbs were similar between the two groups. Two patients' facial nerve size subsided with time, but the decrease in other nerves' sizes were not obvious.

**Conclusion:** Our study showed that serial nerve ultrasound studies are feasible in MFS, and can capture changes in facial nerve size that could complement routine diagnostic tests. Further studies are warranted to determine and compare its test characteristics in MFS.

## Introduction

Miller Fisher syndrome (MFS) is a variant of Guillain-Barre syndrome (GBS)-spectrum disorder, which is a post-infectious monophasic neuropathy (1). The incidence of GBS is between 1.1 and 1.8/100,000/year (2). There are several GBS spectrum disorder variants (acute inflammatory demyelinating polyradiculoneuropathies, acute motor axonal neuropathy, acute motor and sensory axonal neuropathy, and MFS), with distinct geographic distribution of prevalence. The proportion of MFS in the GBS spectrum disorder is higher in Asia (18–26%) than in Western countries (3–5%) (2, 3). The cardinal features of MFS include ophthalmoplegia, ataxia, and hyporeflexia. However, limb paresthesia and facial palsy are not uncommon in pure MFS (1, 4), and MFS could occasionally overlap with another variant of GBS, such as the pharyngeal-cervical-brachial variant (1, 5).

Miller Fisher syndrome and other GBS-spectrum disorders are essentially a clinical diagnosis, while the nerve conduction studies (NCS) and cerebrospinal fluid (CSF) studies often reported non-specific findings in the early phase of GBS-spectrum disorders (6, 7). In routine practice, the laboratory tests are predominantly used to exclude other causes. An objective examination is needed to increase the confidence of a neurologist to diagnose MFS. The contribution of ganglioside antibodies in routine diagnostic setting of GBS and its' variants is limited, as testing for their presence is time consuming, and do not influence treatment early decisions. In addition, availability and performance of different assays tot test for ganglioside antibodies is highly variable. The anti-GQ1b antibody has been reported to be positive in 70–90% of MFS patients (8, 9). In other words, MFS is usually diagnosed by symptoms and neurological examinations.

Magnetic resonance imaging (MRI) had been investigated in GBS, and contrast enhancement had been reported in some studies (10, 11). However, the resolution of MRI was not enough to evaluate the size change in GBS. Recently, morphological studies using sonography have become a popular tool in addition to the classic electrophysiological studies in clinical practice. Nerve enlargement could be clearly seen in entrapment neuropathy with sonography by an experienced operator, but there are few sonography studies on GBS (12–14); those on MFS are even rarer (15). The facial nerve has been investigated by sonography in patients with Bell's palsy (16–19), however, the use of facial nerve sonography has not been explored in MFS before. Therefore, we performed and systematic study to explore sonographic involvement of facial nerves in MFS, and compare this with spinal nerve roots (C5–C7), large arm and leg nerves.

## Materials and Methods

### Subject Recruitment

**Figure 1:**
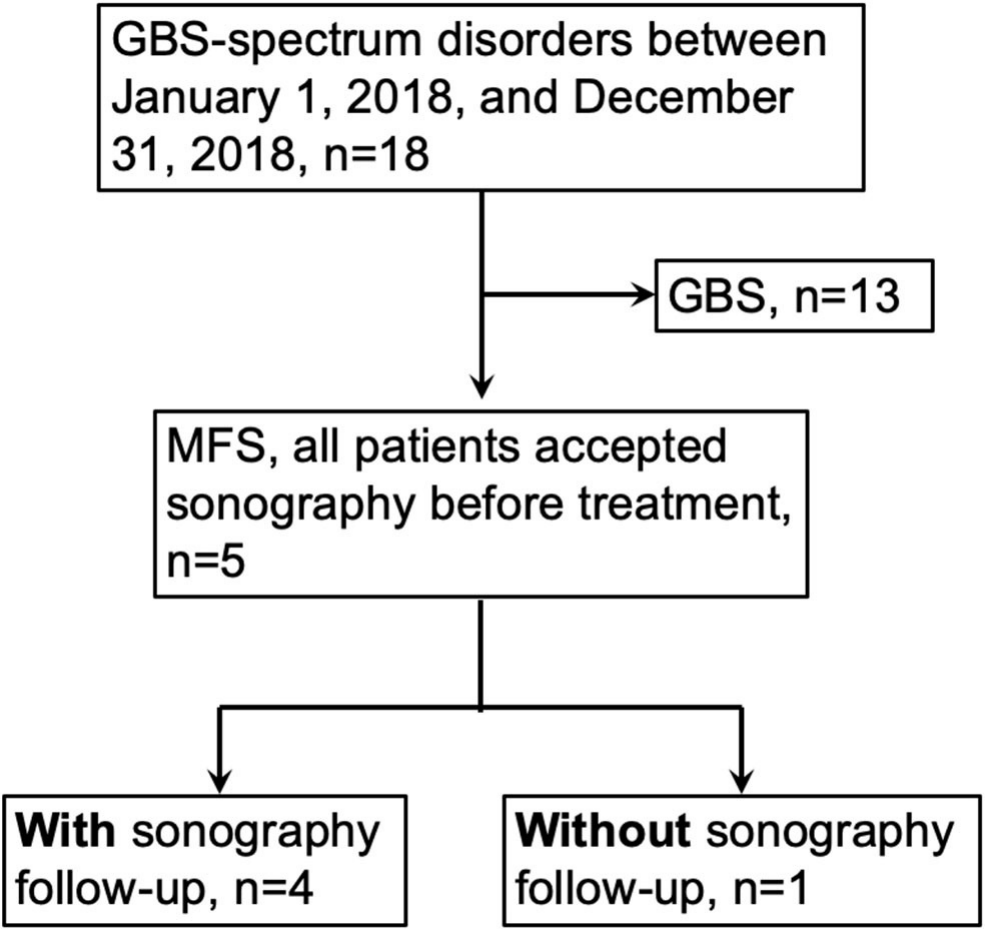
Patient recruitment algorithm. GBS, Guillain-Barre syndrome; MFS, Miller-Fisher syndrome.

This study (201705058RIND) was approved by the Ethics Committee of the National Taiwan University Hospital (NTUH), Taipei, Taiwan, and informed consent was obtained before all procedures were carried out. All procedures performed in studies involving human participants were in accordance with the ethical standards of the institutional and/or national research committee and with the 1964 Helsinki declaration and its later amendments or comparable ethical standards. We used the GBS consensus criteria for diagnosis of MFS: (1) existence of ophthalmoplegia and ataxia with or without areflexia/hyporeflexia; (2) monophasic disease course with an interval between onset and nadir ranging from 12 h to 28 days, followed by a clinical plateau; and (3) alternative diagnosis is excluded. (1) We recruited patients with MFS between January 1, 2018, and December 31, 2018, in our hospital (Figure 1). The inclusion and exclusion criteria of this study were (1) fulfilled the MFS diagnostic criteria mentioned above, (2) accepted the nerve ultrasound study, and (3) no diabetes mellitus, chronic kidney disease, prior Bell's palsy, family history of hereditary diseases, chronic infection, and exposure of toxin. The diagnosis of MFS was made by the primary neurologists, and Dr. HWH was the primary neurologist of case 1, 4, and 5. We recruited the control groups from the colleagues in our department and from the volunteers in our hospital. The neurological examinations were performed by a board neurologist. The inclusion and exclusion criteria of the control group included the following: (1) aged at least 20 years old and <65 years old, (2) no neuropathic symptoms or signs, (3) no abnormality on nerve conduction studies, and (4) no diabetes mellitus, chronic kidney disease, prior Bell's palsy, family history of hereditary diseases, chronic infection, and exposure of toxin.

### Nerve Conduction Studies

Nerve conduction studies were performed with a Nicolet Viking IV Electromyographer (Madison, WI) as per standardized methods in our laboratory (Appendix). The studied nerves included the facial, sural, peroneal, tibial, median, and ulnar (motor and sensory) nerves. Abnormal results in the NCS were defined as reduced amplitudes of compound motor action potentials (CMAPs) or sensory nerve action potentials (SNAPs), conduction block, prolonged distal latencies, slowing of the nerve conduction velocity (NCV), or prolonged minimal F-wave latency (20). The blink reflex study was also performed with recording of R1 and R2 latency. Ipsilateral R1 latency over 13 microseconds, ipsilateral R2 latency over 36 microseconds, or contralateral R2 latency over 38 microseconds was defined abnormal according our own laboratory reference value. The NCS and blink reflex were performed on admission (before treatment), 14 days after first evaluation, and 90 days after first evaluation for the MFS patients.

### Nerve Ultrasound

A nerve ultrasound was performed using 3–12 MHz linear probe with an Affiniti 70G [Philips Medical Instruments, Bothell, WA] by the neurologist (Dr. HWH) in both patient and control groups. The specific MSK mode was not available when the study was performed, so the modes we used (Nerve/Dr. HWH/C Roots mode) were adjusted from the official “Vasc carotid mode” to view the targeted structure most clearly. The examination was not blinded because of the obvious neurological deficit in the MFS group. The cross-sectional area (CSA) was measured with “tracing” methods at the inner border of the hyperechoic rim of the nerve. The CSA was sampled at the nerve of the limbs according to the standardized protocol in our department: median nerve at the wrist, forearm, and elbow; ulnar nerve at the wrist, elbow, and arm; radial nerve at the outlet of the spiral groove; tibial nerve at the medial malleolus; and sural nerve at the lateral malleolus (Appendix). The cervical roots and facial nerves were measured according to previous studies (18, 21). A longitudinal view of the nerve root was observed with the probe placed at the lateral side of the neck. The maximum nerve root width was measured 2 cm distal to the transverse process at the C5, C6, and C7 levels. The nerve of the limbs and spinal nerve was sampled at the dominant hand side. The probe was then placed transversely just beneath the ear lobule to measure the facial nerve inside the parotid gland. The maximal width (inclusion of the hyperechoic border) of the facial nerve was measured according to the previous study (18). The mean width of the facial nerves of both sides was calculated. The duplex signal was checked frequently to prevent mistaking vessels as nerves in all measurements. The ultrasound was also performed on admission (before treatment), 14 days after first evaluation, and 90 days after first evaluation for the MFS patients. All measurements were performed without zoom magnification. In order to evaluate the reliability, we repeated the measurements in all sampled nerves in 10 subjects. The test-retest intra-rater reliability was evaluated by intraclass correlation coefficient, two-way random-effect model, and showed substantial reliability (Appendix).

### Statistical Analysis

Numerical variables were expressed as the mean ± SD and were compared with *t*-tests if the data followed a Gaussian distribution. If the sample size was small, the numerical variables were compared using a non-parametric test (Wilcoxon rank sum test). Fisher's exact test was used to compare categorical data. All analyses were performed using Stata software (StataCorp LP, College Station, TX). The results were considered significant at *p* < 0.05.

## Results

### Clinical, Serological, and Electrophysiological Data

In out hospital, there were 19 patients with GBS-spectrum disorders between January 1, 2018, and December 31, 2018, and there were 5 patients fulfilling the criteria for MFS. All patients presented to our hospital and accepted the thorough examinations within 1 week after symptoms onset. The details of the clinical, serological, and electrophysiological data are summarized in Tables 1, 2. The average age was 53.6 ± 12.9 years, and 3 patients were female. All patients had ophthalmoplegia, ptosis, and ataxia, while facial diplegia and hyporeflexia were noted in 4 patients. Although one patient had hyperreflexia, we still categorized her as MFS rather than overlap with Bickerstaff brainstem encephalitis because of a lack of hypersomnolence (1). In the cerebrospinal fluid study, no patients had pleocytosis, and two patients had elevated total protein. In the electrophysiological study 2 weeks after the disease onset, no abnormality was noted in CMAP, SNAP, and NCV in the nerves of the limbs. There were abnormalities in some parameters, but the abnormality rate was all smaller than 50%: A-wave presence of the lower limbs (*n* = 2), prolonged minimal F-wave latency of the lower limbs (*n* = 1), prolonged R2 latency in the blink study (*n* = 1), and reduced compound motor action potentials in the facial nerve (*n* = 1). Three patients accepted intravenous immunoglobulin, and two patients accepted plasmapheresis. Anti-ganglioside antibodies were checked in four patients, and only two patients had anti-GQ1b IgG antibody. Taken together, our patients presented with core symptoms of MFS, but nearly all had facial weakness and sensory deficits which is infrequent to typical MFS. This particular set of patients likely reflect more severe phenotype of MFS.

**Table 1:** The clinical and anti-GQ1b antibody data for patients with MFS.

**Case/Age/ Sex**	**Precipitating infection**	**MFS key features**			**Ptosis**	**Hypoesthesia**	**Facial diplegia**	**Limb weakness**	**Anti-GQ1b antibody**	**Treatment**	**CSF**		
		**Ophthalmoplegia**	**Ataxia**	**Hyporeflexia**							**Pleocytosis**	Protein (mg/dL)^**^	IgG index^***^
1/65/M	+	+	+	+	+	+	+	–	GQ1b, IgG	IVIg^*^	–	32.8	N/A
2/38/F	+	+	+	+	+	+	+	–	N/A	Plasmapheresis	–	72	0.47
3/65/F	+	+	+	–	+	+	+	–	None	IVIg^*^	–	50.4	0.51
4/41/M	+	+	+	+	+	+	+	+	GQ1b, IgG	Plasmapheresis	–	31.7	N/A
5/56/F	+	+	+	+	+	+	–	+	None	IVIg^*^	–	31	0.42

*MFS, Miller Fisher Syndrome; CSF, cerebrospinal fluid; N/A, not available; IVIg, intravenous immunoglobulin*;

* *IVIg dose: 2 mg/kg*;

** *normal range: 15–45 mg/dL*;

*** *normal range ≦ 0.6*.

**Table 2:** The NCS data for the patients with MFS.

**Case/Age/Sex**		**NCS**	**Blink reflex**	**Facial ENoG**
				
1/65/M	D1	A-wave and prolonged F-wave in bilateral peroneal and tibial nerves	Normal	Normal
	D14	N/A	N/A	N/A
	D90	Normal	Normal	Normal
2/38/F	D1	Normal	Normal	Normal, but smaller CMAP than D14 and D90
	D14	Normal	Normal	Normal
	D90	Normal	Normal	Normal
3/65/F	D1	A-wave in bilateral tibial nerves	Prolonged ipsilateral and contralateral R2 in bilateral stimulation	Normal
	D14	Reduced CMAP in right median and left tibial nerves; A-wave in bilateral peroneal and tibial nerves	Normal	Normal
	D90	Reduced CMAP in right median and left ulnar nerves	Normal	Normal
4/41/M	D1	Normal	Normal	Normal
	D14	Normal	Normal	Reduced CMAP in bilateral facial nerves
	D90	Normal	Normal	Normal
5/56/F	D1	Absent SAP in bilateral median and ulnar nerves; reduced CMAP in right peroneal nerve.	Normal	Normal
	D14	Normal	Normal	Normal
	D90	Normal	Normal	Normal

*NCS, nerve conduction study; Facial ENoG, facial electroneurography; N/A, not available; CMAP, compound motor unit action potential; SAP, sensory action potential*.

### Comparison Between the MFS Group and Control Group Before Treatment

**Figure 2:**
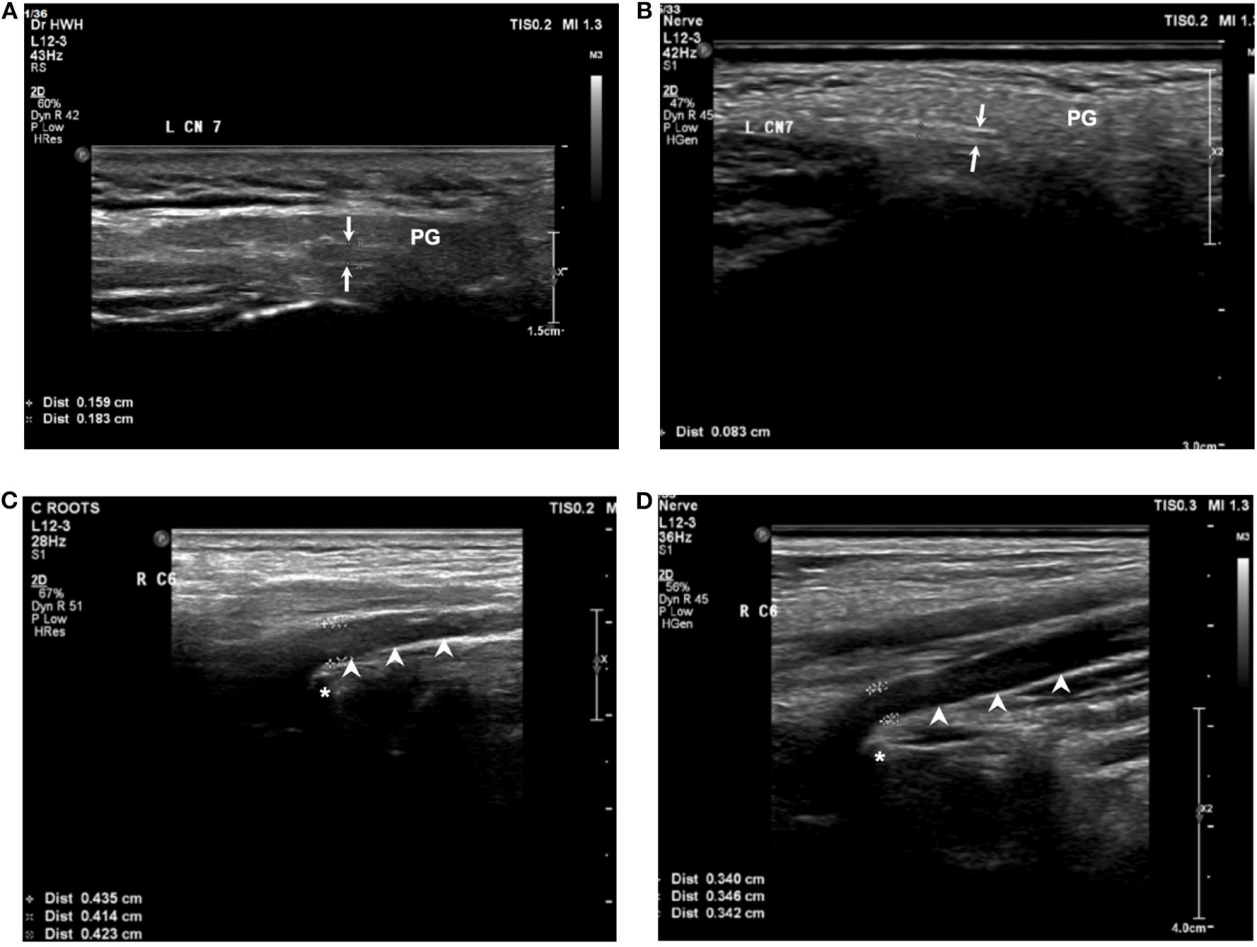
Sonography of facial nerve and cervical root at C6 level for the MFS and normal group at baseline. The width of the facial nerve (arrows) in a patient **(A)** with MFS was larger than in a control **(B)**. The width of the cervical root (arrowheads) at C6 level in a patient with MFS **(C)** and a control **(D)**. PG, parotid gland; asterisk: transverse process of the vertebrae.

All patients underwent sonography before the treatment. However, the facial nerve of one patient (Patient 4) was not sampled because the measurement of facial nerve was not included in the protocol at that time. The spinal nerve at the C5 level was not observed in another patient (Patient 3) because of technical issue. The details of the study and the comparison with the control group are summarized in Table 3. The demographic characteristics were the same between the two groups. The width of the facial nerve in the MFS group was 1.19 ± 0.31 mm, which was significantly larger than in the control group (0.67 ± 0.13 mm, *P* = 0.01) (Figure 2). In the cervical roots, although the width of the cervical roots was larger in the MFS group, no comparisons showed significant differences between the two groups. The CSAs of the nerves in the limbs were similar between the two groups.

**Table 3:** Sonography comparisons between the MFS and normal group at baseline.

	**MFS (*n* = 5)**	**Normal group (*n* = 18)**	***P*-value**
**Demographic characteristics**			
Age	53.6 ± 12.9	58.9 ± 8.1	0.88
Sex (M:F)	2:3	4:14	0.58
Weight (kg)	66.9 ± 16.8	59.4 ± 12.6	0.39
Height (cm)	165.8 ± 9.6	159.5 ± 8.5	0.17
BMI	24.1 ± 4.7	23.3 ± 3.5	0.77
**Facial nerve (mm)**	1.18 ± 0.31	0.67 ± 0.13	0.01
**Cervical roots (mm)**			
C5	3.02 ± 0.22	2.93 ± 0.63	1.00
C6	4.02 ± 0.40	3.45 ± 0.51	0.17
C7	4.03 ± 0.59	3.81 ± 1.01	0.74
**CSA of the nerves in the limbs (mm**^**2**^**)**			
**Median nerve**			
Wrist	10.2 ± 2.13	10.07 ± 2.27	0.92
Forearm	9.76 ± 2.32	8.13 ± 2.6	0.19
Elbow	11.26 ± 3.63	9.96 ± 2.47	0.28
**Ulnar nerve**			
Wrist	4.98 ± 0.78	5.52 ± 1.41	0.26
Elbow	9.93 ± 2.61	8.75 ± 3.2	0.42
Arm	6.5 ± 0.37	7.43 ± 3.0	0.83
Radial nerve	7.23 ± 1.41	4.71 ± 1.15	0.06
Tibial nerve	13.7 ± 3.27	14.5 ± 5.35	0.94
Sural nerve	4.96 ± 2.73	3.5 ± 1.16	0.46

*CSA, cross-sectional area*.

### Serial Follow-Up

**Figure 3:**
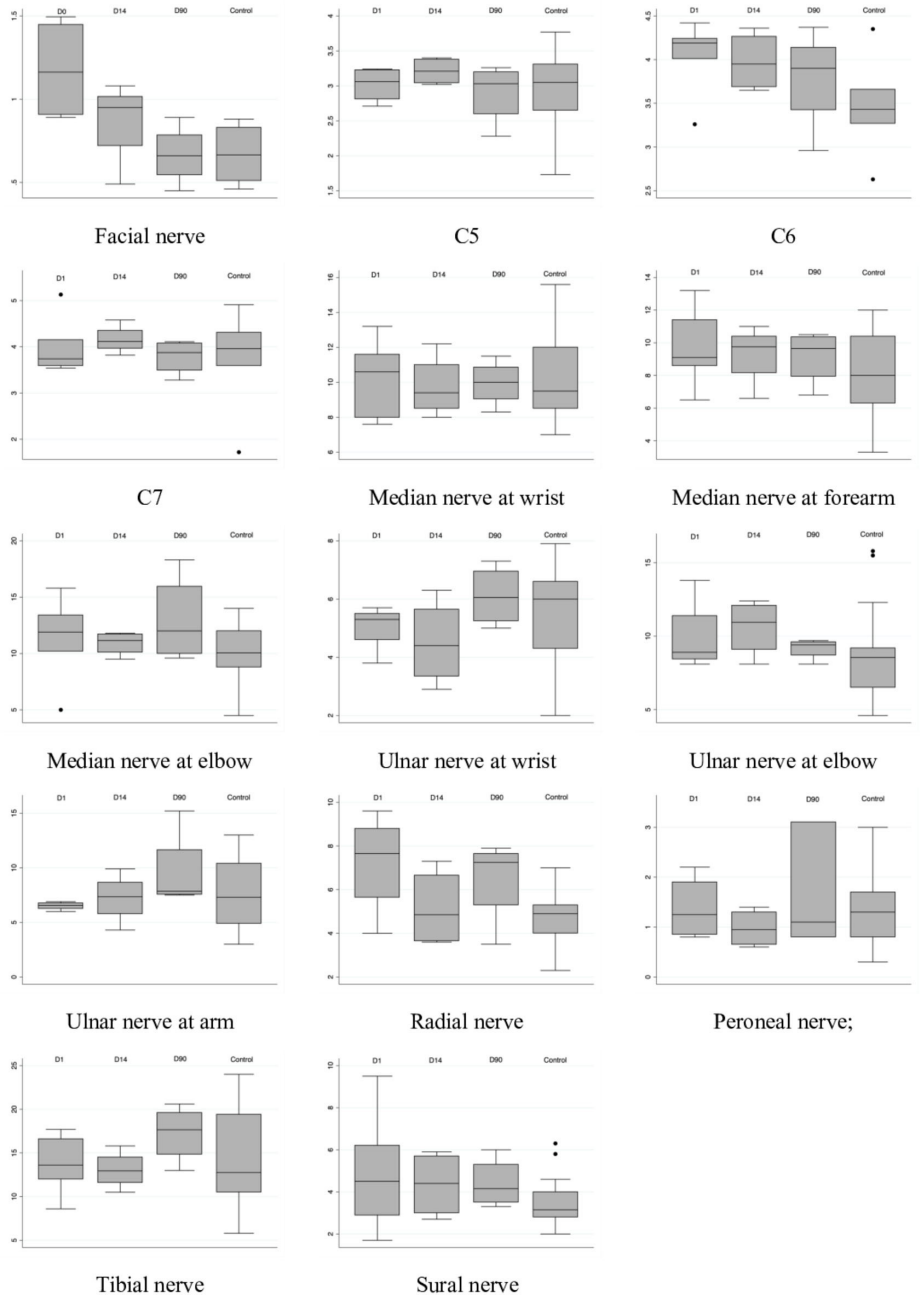
Serial nerve sonography studies for patient with MFS and control group.

**Figure 4:**
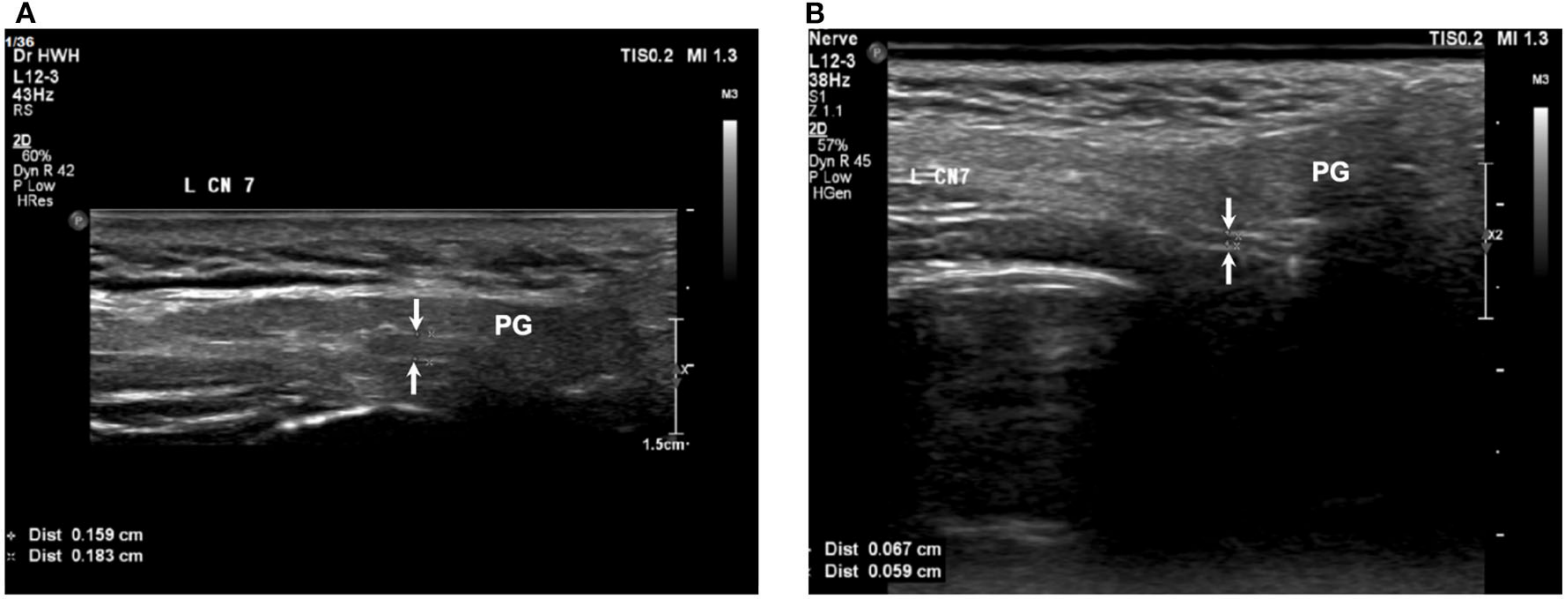
The facial nerve size subsided with time in a patient with MFS. **(A)** Before treatment, **(B)** 90 days after treatment. Arrows: facial nerve; PG, parotid gland.

The symptoms improved gradually in all patients, and the above abnormal findings in the electrophysiological study also improved in all patients by the follow-up examination 3 months later. Four patients underwent serial follow-up with sonography. The serial sonography data are summarized in Figures 3, 4. The facial nerve size decreased with time in 2 patients, but one patient was similar in the follow-up, though that patient's nerve size was within normal limits at the disease onset.

## Discussion

This study demonstrated the presence of an enlarged facial nerve in patients of MFS. The facial nerve width decreased in parallel with the clinical symptoms in two of our patients.

There is only one study exploring ultrasound in MFS patients. In 2015, Decard et al. reported two cases of MFS with enlargement of the vagus nerve, spinal nerves, and/or peripheral nerves (15). The enlargement improved 2 weeks later, and the clinical symptoms also improved. The enlargement of the cervical root in patients with MFS indicated involvement of nerves other than brain and cranial nerves, which may explain the generalized hyporeflexia in MFS. We proposed the nerve in four limbs may be involved in MFS even the symptom is not obvious. Because hyporeflexia is in the diagnostic triad of MFS and distal limb numbness is often noted in patient with MFS, we thought the peripheral nerves or nerve root should also be involved. A thorough nerve ultrasound study with specific ultrasound scores (such as UPSS-ultrasound pattern sum score) could help diagnose different diseases (22, 23). However, the facial nerve was not evaluated in the previous studies, so we included the facial nerve and spinal nerve into our protocol. Notably, the spinal nerve size was not significantly different between the two groups in our study. To explain the different results, we compared the patient characteristics and methodology between our study and Decard's report. In view of patient characteristic, both their patients had limb weakness, and was classified as MFS-plus variant of GBS, and only two patients in our group had limb weakness. The age is also older in our group (Our patient group: 53.6 ± 12.9 vs. Decard's reports: 46 and 17 year-old). In methodology, both of us followed the same protocol (21, 24). Actually, the diameter of spinal nerve of C5 and C6 was not obviously different between patients of Decard's group and ours. Instead, the value from our control group was markedly larger than the reference value of spinal nerve of Decard's group. The reference value of spinal nerve of Decard's group was derived from Japanese adults, and Decard's group is from Europe (The race was not mentioned in their report) (15, 24). If we compared our control group with the Japanese adults' normal value, the age is markedly order than Japanese reference values (Our control group: 53.6 ± 12.9 vs. Japanese adults: 35.4 ± 9.7), while there was no obvious difference in height, weight, and BMI (24). As a result, age, clinical symptoms, and races may serve as an important factor for the different results.

MRI occasionally shows enhancement of the cranial nerve in patients with MFS, but the resolution of MRI makes the evaluation of nerve enlargement challenging. The resolution of ultrasound makes the quantitative evaluation of nerve enlargement possible. Several studies have evaluated the facial nerve in patients with Bell's palsy by ultrasound (16, 17, 19). Tawfik et al. reported an enlarged facial nerve in patients with Bell's palsy compared with controls and a significant side-to-side difference as well (17). Facial nerve ultrasound had not been studied in patients with MFS previously. Our study is the first to demonstrate the use of facial nerve ultrasound in GBS-spectrum disorders.

In contrast to MFS, GBS and CIDP have been studied with sonography. CIDP is a chronic inflammatory polyneuropathy with an “onion bulb” formation on pathology (25). Repetitive demyelination and remyelination make the nerve enlarged and can be observed by MRI or ultrasound. The focal enlargement, especially on the non-entrapment site and proximal portion of the nerves and spinal nerves, could be clearly observed on ultrasound and is very different from the presentation of usual entrapment neuropathy (26, 27). Besides, the cranial nerve hypertrophy had been reported in patients with CIDP, but only being studied by MRI. Our study provided an easy, inexpensive and non-invasive way to evaluate the facial nerve in CIDP at both relapse and remitting state. In GBS, enlargement was observed in different parts of the nerve, and the enlargement subsided in parallel with the clinical symptoms (13, 28). In other words, the nerve size decreased as the symptoms decreased. Interestingly, several studies reported the enlargement of the cervical spinal nerve at the early phase of GBS, and it may reflect the radiculopathy pattern in NCSs in the early phase of GBS (13). However, the nerve enlargement in GBS was smaller than in CIDP (29), suggesting the different pathogenesis underlying the nerve enlargement in CIDP and GBS. Besides, facial nerve involvement was frequently noted in clinical presentation and by MRI in patients with GBS, but facial nerve hypertrophy was not reported before. This may be related to that MRI resolution is not enough to show the facial nerve enlargement in GBS.

The pathogenesis underlying the facial nerve enlargement may be related to nerve edema or “onion-bulb” formation. Because remyelination takes times, early nerve enlargement and improvement within months in GBS-spectrum disorders should be related to edematous changes rather than an “onion-bulb” formation. The prognosis is good in most MFS patients, so biopsy or autopsy studies are rare. Instead, pathology of GBS had been investigated widely by sural nerve biopsy or autopsy. In addition to patchy demyelination and perivascular mononuclear inflammatory infiltration throughout the peripheral nerve system, endoneurial and subperineural edema were also noted in acute inflammatory demyelinating polyneuropathy (AIDP). In acute motor axonal neuropathy/acute motor and sensory axonal neuropathy (AMAN/AMSAN), primary axonal degeneration with a paucity of inflammatory lymphocytic infiltration is the main pathology finding, and edema is less frequently reported (25, 30). One sonographic study showed that the nerve root width was significantly larger in an AIDP group than in patients with AMAN and unclassified conditions (14), which also supported the nerve edema should be related to the nerve enlargement. Because sonographic study of GBS is still limited and the patient number is small in each study, further studies integrating clinical, electrophysiological, imaging, and pathology evaluations are needed.

There were some limitations to our study. First, facial palsy is not the cardinal feature of MFS, and there was only one patient without facial palsy in our cohort. However, the spinal nerve enlargement and multiple cranial nerve enhancements on MRI in patients without clinical symptoms (31, 32) suggest that the nerve could be involved without symptoms. Further study of MFS patients without facial palsy could clarify the usefulness of facial nerve ultrasound. Second, the sonography was performed by a single operator (Dr. HWH) who was not blinded to the diagnosis of the patients. The single operator prevents the inter-rater variation, but the open-label study makes the measurement having some bias. An operator blinded to the diagnosis could ameliorate the bias, but the examination is still hard to be totally blinded for an experienced neurologist because of the obvious symptoms of MFS. In addition, the reliability of nerve sonography is not evaluated, and these should be evaluated in the future study. A disease control group (such as Bell's palsy, CIDP, and Charcot-Marie-Tooth disease) could also help optimize the nerve sonography. Third, the nerve ultrasound study was performed with a relatively low frequency range (3–12 MHz) probe. The low frequency range probe still could see the superficial structure but with poorer resolution, but it could depict the spinal nerves more clearly than pure high frequency probe (>15 MHz). The better way is buying a wide rage frequency probe (such as L5–18 or L4–18 MHz probes in Philips Affinity 70) or two probes with different frequency ranges, but it is not available in our department. Instead, we chose the lower frequency probe for covering all structures we want to study. Our ultrasound mode was not an official musculoskeletal mode, but a mode derived from the official “Vasc carotid mode,” whose setting was adjusted by the Philip official technician. Fourth, the study didn't include the disease control group to see whether facial nerve enlargement is specific to MFS. We considered the facial nerve enlargement is not specific to MFS, because previous studies on Bell's palsy also reported enlarged facial nerve (16, 17, 19). Last, the number of patients is small and the symptoms are not homogeneous, and some patients had overlap with GBS. The GBS is a rare disease, and the MFS is even more rare. It is hard to recruit enough patients to achieve the statistic power in a single hospital. We used a non-parametric test, which is more strict than parametric test to perform the statistics. The small number may also explain the discrepancy in the spinal nerve width between our study and the previous report (15). Besides, an extensive sonography study including facial nerve, spinal nerves and nerve of the limbs would clarify the nerve involvement in MFS, whereas the generalized hyporeflexia is the cardinal feature. Multicenter and international collaboration is needed for investigating sonography use in MFS/GBS, but also for the prediction of clinical course and treatment response.

## Conclusion

Sonography revealed an enlarged facial nerve at the onset of MFS, which decreased 3 months after the disease onset. Further validation is needed to confirm nerve ultrasound as an objective tool to evaluate MFS.

## Data Availability Statement

The datasets generated for this study are available on request to the corresponding author.

## Ethics Statement

The studies involving human participants were reviewed and approved by Ethics Committee of the National Taiwan University Hospital, Taipei, Taiwan. The patients/participants provided their written informed consent to participate in this study.

## Author Contributions

H-WH, C-CC, and S-TH conceived and planned the studies and wrote the manuscripts with input from all authors. All authors contributed to data acquisition and analysis.

## Conflict of Interest

The authors declare that the research was conducted in the absence of any commercial or financial relationships that could be construed as a potential conflict of interest.
