# Supplementary material for: A Pilot Study on Serial Nerve Ultrasound in Miller Fisher Syndrome
Source: Front Neurol. 2020 Aug 14;11:865. doi: 10.3389/fneur.2020.00865 (PMC7457056; doi:10.3389/fneur.2020.00865)
Supplement: Supplementary file 1 [file Table_1.DOCX]

**Nerve conduction study protocol:**

1. Nerve conduction study

The study included motor and nerve nerves.

1. Motor nerve: The recording electrode (G1) was placed over the muscle belly and the reference electrode (G2) was placed over the tendon of recording muscle. The ground electrode was placed near the stimulation site. The stimulation at the distal site was 8cm proximal to the G1 electrode. The stimulation at the proximal was defined as below. The motor NCV was calculated as distance (between two stimulation site) divided by the latency difference (between two stimulation site):

|  | Record muscle | Distal stimulation | Proximal stimulation | Normal value |
| --- | --- | --- | --- | --- |
| Median nerve | abductor pollicis brevis | Wrist, 8cm proximal to G1 | Elbow | DL: < 4.2 ms  CMAP: > 4.6 mV  NCV: > 50m/s |
| Ulnar nerve | ADM | Wrist, 8cm proximal to G1 | 1. Elbow, below cubital tunnel 2. Arm, above the cubital tunnel | DL: < 3.5 ms  CMAP: > 6 mV  NCV: > 50m/s |
| Peroneal nerve | Extensor digitorum brevis | Ankle (anterior side), 8cm proximal to G1 | Fibular head | DL: < 5.5ms  CMAP: > 2 mV  NCV: > 40m/s |
| Tibial nerve | Abductor hallucis | Ankle (medial side), 8cm proximal to G1 | Popliteal fossa | DL: < 5.5ms  CMAP: > 2 mV  NCV: > 40m/s |
| DL: distal latency; CMAP: compound muscle action potential; NCV: nerve conduction veleocity | | | | |

1. Sensory nerve: The sensory study was performed antidromically. The recording electrode (G1) was placed over the muscle tendon or joint, and the reference electrode (G2) was placed 3cm distal to the G1. The ground electrode was placed near the stimulation site. The stimulation at the distal site was 14 cm proximal to the G1 electrode. The sensory NCV was calculated as distance (between the stimulation site and G1) divided by the latency difference (between the stimulation site and G1). The de

|  | Record site (G1) | Distal stimulation | Normal value |
| --- | --- | --- | --- |
| Median nerve | Metacarpophalangeal joint of index finger | Wrist, 14 cm proximal to G1 | SAP: > 10 μV  NCV: > 50m/s |
| Ulnar nerve | Metacarpophalangeal joint of 5^th^ finger | Wrist, 14 cm proximal to G1 | SAP: > 8.5 μV  NCV: > 50m/s |
| Sural nerve | Extensor digitorum brevis | Lateral shin, 14cm proximal to G1 | SAP: > 5 μV  NCV: > 50m/s |
| SAP: sensory action potential; NCV: nerve conduction velocity. | | | |

1. F-wave

The recording electrode (G1), reference electrode (G2), and ground electrode are placed as motor nerve conduction study. The stimulation site is also similar as distal stimulation site of motor nerve conduction study. Ten F-wave responses were recorded in each nerve to determine minimal F-wave latency and existence of A-waves. The minimal F-wave latency is abnormal if over 30 ms in nerve s of upper limbs and 50 ms in nerves of lower limbs.

1. Blink reflex

The blink reflex study was performed with recording of R1 and R2 latency. The recording site is over orbicularis oculi muscle, at the medial lower eyelid. The reference electrode was places over the orbicularis oculi muscle, at the lateral surface of the eye. The ground electrode is placed over the chin. The stimulation site was at the supraorbital nerve, at the supraorbital notch. Supramaximal stimulation was performed at either side respectively. Ipsilateral R1 latency over 13 microseconds, ipsilateral R2 latency over 36 microseconds, or contralateral R2 latency over 38 microseconds was defined abnormal according to our own laboratory reference value.

1. ENoG

The ENoG study was performed with recording of CMAP of facial nerve. The recording site was the G1 electrode over the ipsilateral nasolabial fold, and the G2 electrode over the contralateral nasolabial fold. The ground electrode is placed in the center of forehead. The stimulation site was at the cheek near the ear (stylomastoid foramen). The CMAP below 1mV was defined abnormal at our lab.

**Ultrasound protocol:**

Upper limb:

**Median nerve**

1. Wrist: The patient put the hand at neutral position. Measure at the wrist crease, when seeing the tendon of FCR (flexor carpi radialis) and scaphoid bone. The median is also under transverse carpal ligament.
2. Forearm: Track the median nerve proximally from the wrist. When seeing the pronator quardratus muscle disappeared, measure the CSA. The pronator quadratus muscle is the only muscle with transverse axis of the distal arm.
3. Elbow: draw a line between the medial and lateral epicondyle, measure the smallest CSA at the line.

**Ulnar nerve**

1. Wrist: The patient at neutral position as measuring the median nerve. After measuring the median nerve, move the probe horizontally to measure the ulnar nerve at the same level.
2. Elbow: The patient flex the elbow in 90 degree. Then measure the ulnar nerve at the cubital tunnel, and the medial epicondyle should be seen at the same time.
3. Arm: in the middle of upper arm.

**Radial nerve**: The patient pronate the hand and put it on the table. Measure the CSA when the radial nerve just leaves the spiral groove (leave the humerus bone). Do power duplex scan to prevent measure the radial artery rather than radial nerve.

Lower limb

**Peroneal nerve:** The patient put the leg at neutral position, draw a line between the medial and lateral epicondyles, measure the CSA at the line. Sweep the probe to and fro to see the peroneal nerve. See the nerve besides the anterior tibial artery. The nerve may not be seen because of the tiny size.

**Tibial nerve**: put the probe just above the medial malleolus of tibia bone. Measure the smallest CSA.

**Sural nerve:** put the probe just above the lateral epicondyle of tibia bone. See the nerve beside the small saphenous vein. Measure the smallest CSA without compression of the small saphenous nerve.

**Evaluation of intra-rater reliability of nerve ultrasound**

All image measurements were performed by a single examiner (HW, Hsueh) to avoid inter-rater variation.

In order to evaluate the intra-rater reliability, we performed rating and re-rating on the 130 nerves (13 sample sites, 10 subjects). The test-retest intra-rater reliability was evaluated by intraclass correlation coefficient, two-way random-effects model.

|  | Mean ICC (95% CI) | P-value |
| --- | --- | --- |
| Facial nerve | 0.77 (0.02-0.94) | 0.024 |
| C5 | 0.88 (0.43-0.97) | 0.005 |
| C6 | 0.95 (0.83-0.99) | <0.001 |
| C7 | 0.93 (0.59-0.99) | <0.001 |
| Median nerve |  |  |
| Wrist | 0.86 (0.47-0.96) | 0.003 |
| Forearm | 0.89 (0.53-0.97) | 0.002 |
| Elbow | 0.96 (0.82-0.99) | <0.001 |
| Ulnar nerve |  |  |
| Wrist | 0.88 (0.49-0.97) | 0.003 |
| Elbow | 0.91 (0.65-0.98) | 0.001 |
| Arm | 0.98 (0.93-0.99) | <0.001 |
| Radial nerve | 0.95 (0.82-0.99) | <0.001 |
| Tibial nerve | 0.86 (0.42-0.96) | 0.005 |
| Sural nerve | 0.92 (0.71-0.98) | <0.001 |

ICC: Intraclass correlation coefficient
